# Identification of linderalactone as a natural inhibitor of SHP2 to ameliorate CCl_4_-induced liver fibrosis

**DOI:** 10.3389/fphar.2023.1098463

**Published:** 2023-02-09

**Authors:** Yi Zhang, Binhao Cai, Yingying Li, Ying Xu, Yuhan Wang, Lulu Zheng, Xiaochun Zheng, Lina Yin, Gaozhi Chen, Yunxiang Wang, Guang Liang, Lingfeng Chen

**Affiliations:** ^1^ Affiliated Yongkang First People’s Hospital and School of Pharmacy, Hangzhou Medical College, Hangzhou, Zhejiang, China; ^2^ Chemical Biology Research Center, School of Pharmaceutical Sciences, Wenzhou Medical University, Wenzhou, Zhejiang, China; ^3^ Department of Pharmacy, Tongde Hospital of Zhejiang Province, Hangzhou, Zhejiang, China; ^4^ Department of Pharmacy, Zhejiang Provincial People’s Hospital, Affiliated People’s Hospital, Hangzhou Medical College, Hangzhou, Zhejiang, China

**Keywords:** Src homology 2 domain-containing phosphatase 2, liver fibrosis, high-throughput screening, linderalactone, natural products

## Abstract

Liver fibrosis is characterised by the activation of hepatic stellate cells (HSCs) and matrix deposition. Accumulating evidence has revealed that the oncogenic protein tyrosine phosphatase Src homology 2 domain-containing phosphatase 2 (SHP2) acts as a therapeutic target of fibrosis. Although several SHP2 inhibitors have reached early clinical trials, there are currently no FDA-approved drugs that target SHP2. In this study, we aimed to identify novel SHP2 inhibitors from an in-house natural product library to treat liver fibrosis. Out of the screened 800 compounds, a furanogermacrane sesquiterpene, linderalactone (LIN), significantly inhibited SHP2 dephosphorylation activity *in vitro*. Cross-validated enzymatic assays, bio-layer interferometry (BLI) assays, and site-directed mutagenesis were used to confirm that LIN directly binds to the catalytic PTP domain of SHP2. *In vivo* administration of LIN significantly ameliorated carbon tetrachloride (CCl_4_)-induced HSC activation and liver fibrosis by inhibiting the TGFβ/Smad3 pathway. Thus, LIN or its derivatives could be considered potential therapeutic agents against SHP2-related diseases, such as liver fibrosis or NASH.

## 1 Introduction

Hepatic fibrosis is a dysregulated wound-healing pathological feedback resulting from a broad range of chronic liver diseases, including alcoholic liver disease, non-alcoholic fatty liver disease (NAFLD), viral infection, and autoimmune liver diseases (Roehlen et al., 2020). Liver fibrosis has severe complications, including portal hypertension and liver failure, liver cirrhosis, and hepatocellular carcinoma (Kisseleva and Brenner, 2021). Hepatic stellate cells (HSCs) are central drivers of liver fibrosis progression. Quiescent HSCs are located in the space of Disse between the sinusoidal endothelium and the hepatocytes. In response to toxic injury, HSCs are activated and differentiate into α-smooth muscle actin (α-SMA)-expressing myofibroblasts, which then generate extracellular matrix (ECM) proteins. Additionally, large amounts of cytokines and chemokines secreted by activated HSCs drive hepatic inflammation and promote fibrogenesis (Tsuchida and Friedman, 2017; Ezhilarasan et al., 2018). Thus, preventing or reverting HSC activation will effectively decelerate hepatic fibrosis development. Despite the continued discovery of novel molecular mechanisms and mediators, there are currently no approved agents for liver fibrosis. Therefore, there is an enormous unmet clinical need for anti-fibrotic therapies to prevent and treat hepatic fibrosis.

**FIGURE 1:**
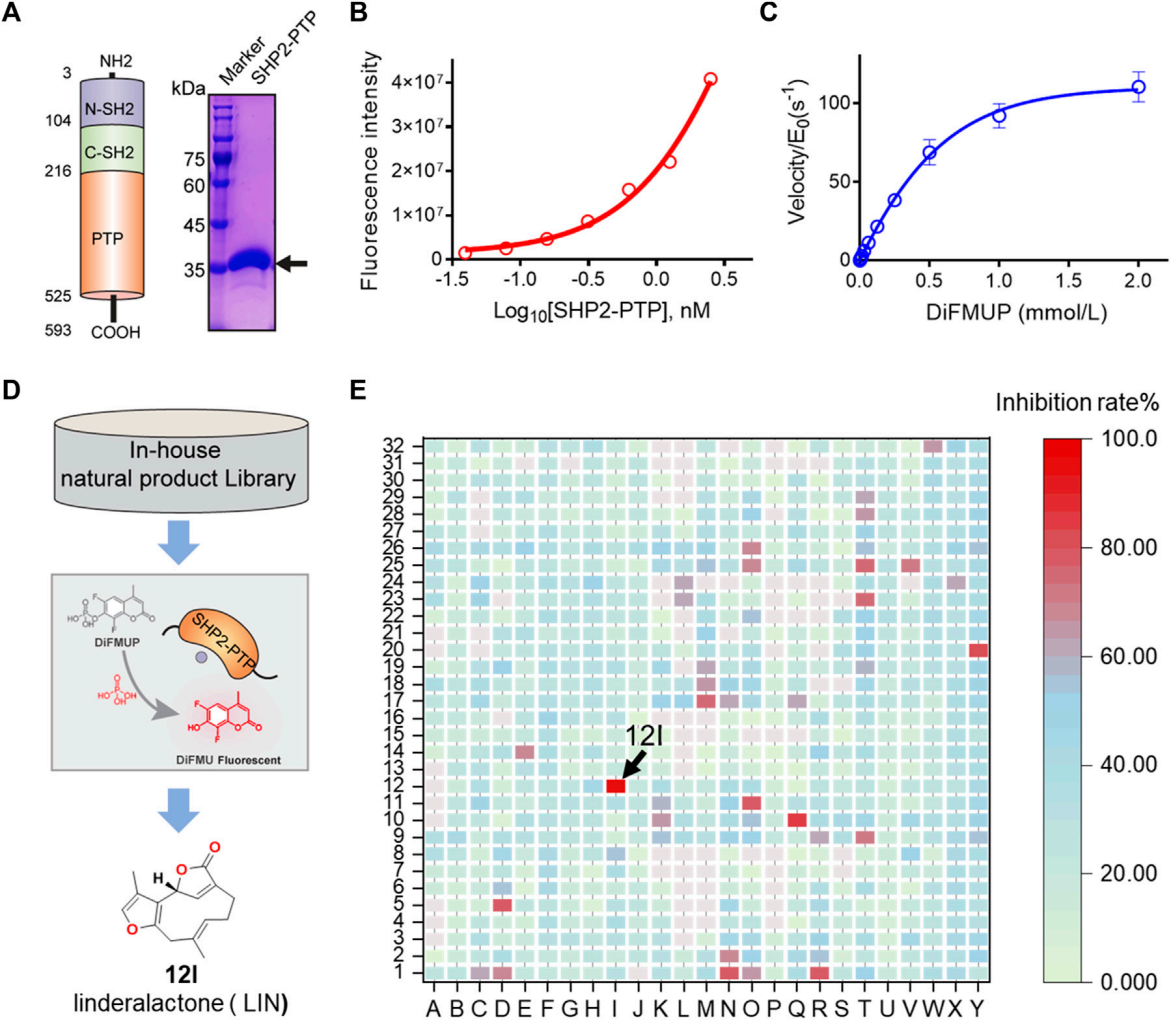
HTS and identification of novel SHP2 inhibitors from natural product library. **(A)** Schematic illustration of three SHP2 subdomains containing N-SH2, C-SH2, and PTP domain shown in blue, green, and orange, respectively. The PTP domain of SHP2 was expressed and purified, and the purity of the his-tagged labelled SHP2 PTP domain are evaluated by SDS-PAGE and Coomassie blue staining with a molecular weight of approximately 38.0 kDa. **(B)** The dephosphorylation activity of rhSHP2-PTP with the protein concentrations ranging from 0 to 2.50 nM. **(C)** Enzyme kinetics of substrate DiFMUP dephosphorylation by SHP2-PTP. **(D,E)** Drug screening paradigm for SHP2 inhibitor HTS from an in-house natural product library (800 compounds) using the DiUFMP assay, leading to the identification of linderalactone (12I) as a novel SHP2 inhibitor.

Protein tyrosine phosphatase (PTP), Src homology 2 domain-containing phosphatase 2 (SHP2), was the first oncogenic molecule to function as a signal transducer for receptor tyrosine kinases (RTKs) (Chan and Feng, 2007). SHP2 is ubiquitously expressed in cells and regulates cell survival, proliferation, and migration through the RAS-ERK, JAK-STAT, PI3K-AKT, and programmed cell death 1 (PD-1) immune checkpoint pathways (Okazaki et al., 2013; Chen et al., 2016). Aberrant SHP2 activation is associated with various cancer types, including hepatocellular carcinoma (Yuan et al., 2020; Song et al., 2021). SHP2 is composed of a single PTP domain and two tandem Src homology 2 (SH2) domains (Figure 1A). (Hof et al., 1998) In the resting state, SHP2 activity is strictly controlled by its autoinhibition mechanism. Upon binding of the phosphorylated substrate, SHP2 is altered from an autoinhibited state to an activated state (LaRochelle et al., 2018). Recently, an increasing number of studies have reported that SHP2 is closely associated with fibrosis (Miao et al., 2021). SHP2 expression was found to be increased in patients with liver cirrhosis compared to healthy controls. Pharmacological inhibition or genetic knockout of SHP2 downregulates STAT3 activation through JAK2 dephosphorylation, thereby ameliorating TGFβ-induced fibrosis (Zehender et al., 2018). Furthermore, SHP2 inhibition was reported to downregulate the phosphorylation of platelet-derived growth factor receptor α (PDGFRα) derived from HSCs, thus reducing liver fibrosis (Kostallari et al., 2018). Additionally, SHP2 in HSCs promotes its pro-fibrotic effect by enhancing the release of fibrogenic extracellular vesicles. Deletion of HSC-derived SHP2 reduced CCl_4_-induced liver fibrosis in a mouse model (Gao et al., 2020). Thus, SHP2 is closely related to the activation of HSCs and is therefore an attractive novel therapeutic target for liver fibrosis.

Natural products (NPs) are an abundant source for the identification of bioactive skeletons owing to their great structural diversity (Atanasov et al., 2021). Historically, over 30% of FDA-approved drugs are directly used or structurally modified from NPs. Currently, NPs are becoming increasingly recognised as an applicable therapy for fibrosis (Li et al., 2019; Newman and Cragg, 2020). For instance, tanshinone IIA and ligustrazine show therapeutic, antifibrotic effects in clinical studies (Chen et al., 2018; Zhang et al., 2018). Hence, identification the molecular target of NPs was important for the discovery of novel antifibrotic agents. During the past two decades, NPs have been demonstrated to be ideal lead compounds for the development of PTP inhibitors. Several natural product-derived compounds have been developed as potent PTP1B antagonists (Johnson et al., 2002; Jiang et al., 2012). Therefore, identifying active NPs which can inhibit the SHP2-mediated HSCs activation and fibrosis are of great importance.

In this study, to identify novel SHP2 inhibitors for liver fibrosis therapy, we developed a cross-validated high-throughput screening (HTS) DiFMUP assay platform, leading to the identification of Linderalactone (LIN) as a novel SHP2 inhibitor from the NP library. Subsequently, molecular docking, BLI, and site-directed mutagenesis experiments were performed to validate the direct binding site of LIN to SHP2. The effects of LIN on HSCs activation and liver fibrosis were investigated *in vivo*.

## 2 Materials and methods

### 2.1 Protein expression and purification

Human SHP2 PTP domain (A237–I529) expression constructs were engineered by cloning *PTPN11* (NP_002825.3) into the pET30 plasmid with a His-tag to support further protein purification. The K366A/Q510A double mutation was introduced into the wild-type template using a site-directed mutagenesis kit (E0554S; New England Biolabs). The expression constructs were sequenced and transformed into BL21 (DE3)-competent cells. *E. coli* cells were grown at 37°C in an LB culture medium in the presence of 100 μM mL^−1^ kanamycin until the OD_600_ reached 1.0. The medium was then cooled to 18°C and 1 mM isopropyl ß-D-1-thiogalactopyranoside (IPTG) was added to induce protein expression overnight.

Cell pellets were collected and suspended in a buffer of 50 mM Tris-HCl (Ph = 8.5), 150 mM NaCl supplemented with 10% glycerol, and lysed by homogeniser (JN-BIO, China). After centrifugation, the collected supernatants were diluted with a Tris-HCl buffer (pH = 8.5) containing 50 mM imidazole and 150 mM NaCl and then loaded onto a Ni-NTA column (#88221, Thermo). The bound SHP2 protein was eluted with a buffer containing 150 and 300 mM imidazole. The pooled protein was loaded onto a HiTrap Q column after dilution in 25 mM Tris-HCl buffer. After elution using a linear gradient of NaCl (0–0.5 M), peak fractions containing the SHP2 protein were concentrated and applied to a gel filtration Superdex 200 column (GE Healthcare). Purified SHP2-PTP proteins were flash-frozen and stored. Similar expression and purification protocols were used for the expression and purification of the full-length SHP2 and SHP2-PTP ^K366A/Q510A^ double mutants.

### 2.2 SHP2 inhibition assay

A surrogate phosphatase substrate, 6,8-difluoro-4-methylumbelliferyl phosphate (DiFMUP, Thermo Fisher, #D22065), was used to monitor the catalytic activity of SHP2 in a prompt fluorescence assay format. Specifically, the DiFMUP phosphatase assay was performed at 20°C in a 384-well Griener CELLSTAR black polystyrene plate in the buffer condition of 60 mM HEPES (pH7.5), 75 mM NaCl, 75 mM KCl, 1 mM EDTA, 0.05% Tween-20, 5 mM dithiothreitol (DTT). The final reaction volume was 25 μL. NP compound libraries were obtained from TargetMol at a stock concentration of 10 mM, dissolved in DMSO. The purity of the active compound LIN used in this study is 96.27% as tested by HPLC analysis. The HPLC traces is shown in Supporting Information. We co-incubated 0.5 nM of SHP2-PTP enzyme with tested NPs for 30 min at 20°C, followed by the addition of DiFMUP into the reaction. After 30 min, 5 μL of bpV(phen) solution was added to quench the dephosphorylation reaction. The signals were read on a plate reader (SpectraMax iD5, Molecular Devices). The excitation and emission wavelengths were 340 and 450 nm, respectively. The inhibitor dose-response curves were evaluated using a normalised IC_50_ regression curve fitting with control-based normalisation. For the full-length SHP2 inhibition assay, 0.5 μM of a bisphosphorylated pIRS-1 peptide (ChinaPeptides Ltd. Shanghai) was added for partial SHP2 activation.

### 2.3 BLI assay

The BLI assay was performed using a FortéBio Octet 96 system. To label the biotin group on SHP2 proteins, NHS-PEG12-Biotin (Thermo Fisher, 21312) was added to the purified SHP2-PTP solution (Thermo Fisher, 21312) at 1.5:1 ratio for 2 h at 4°C. The reaction mixture was then loaded onto a column (GENEMORE, #G-MM-IGT) to remove excess free biotin. The biotin-labelled-SHP2-PTP protein was loaded onto super streptavidin (SSA) sensors (#18-5057, Octet) in a buffer containing 200 μL 25 mM HEPES, 150 mM NaCl, and 0.02% (v/v) Tween-20. After equilibrium, the kinetics of the LIN and SHP2-PTP association were analysed by soaking the SSA sensors in a compound solution with various LIN concentrations for 300 s (6.25, 12.5, 25, 50, 100, and 200 μM), followed by 300 s of dissociation in the same HEPES buffer. The equilibrium constant (KD) values were assessed using the FortéBio data analysis software by fitting the kinetic data using a 1:1 binding model.

### 2.4 Molecular docking study

The binding mode between SHP2 and LIN was analysed using the *Glide* module in the *Schrödinger* package. The SHP2 structure (PDB ID:4RDD) and LIN were processed using the *Protein Preparation Wizard* and *LigPrep* modules in the *Schrödinger* package (Fodor et al., 2018). Grids were generated for PTP binding sites using *the Receptor Grid Generation* module in the *Schrödinger* package. The *Glide* module of the *Schrödinger* package was used to generate the predicted binding positions between LIN and SHP2.

### 2.5 Cell culture

The LX-2 cell line was purchased from the Cell Bank Type Culture Collection of the Chinese Academy of Sciences (Shanghai, China). Cells were cultured in DMEM supplemented with 10% [v/v] foetal bovine serum, 2 mM glutamine, 100 U/ml penicillin, and 100 mg/mL streptomycin. SHP-2 and control siRNAs were obtained from Suzhou GenePharma Co., Ltd. (sense 5'-3': GAG​AGA​GGA​AAG​AGU​AAA​UTT; antisense 5'-3': AUU​UAC​UCU​UUC​CUC​UCU​CTT). Transfection was performed according to the manufacturer’s instructions. Subsequent real-time PCR and western blotting were performed to analyse transfection efficiency.

### 2.6 Animal model and treatment

C57BL/6 mice (20 ± 2 g) were obtained from Hangzhou Medical College Experimental Animal Center (Hangzhou, China). The animals were maintained under controlled temperature (22°C ± 1°C), humidity (50%), and light (12 h light/12 h dark). The animals were fed a standard laboratory diet and provided with free access to tap water. All animals received humane care according to institutional animal care guidelines approved by the Experimental Animal Ethical Committee of Hangzhou Medical College. Forty mice were randomly divided into six groups: (1) vehicle control (*n* = 7); (2) CCl_4_ model (*n* = 7); (3) CCl_4_ + LIN (20 mg/kg) (*n* = 7); (4) CCl_4_ + LIN (40 mg/kg) (*n* = 7); (5) LIN (40 mg/kg) (*n* = 7); and (6) CCl_4_ + silymarin (SIL) (200 mg/kg) (*n* = 5). LIN and SIL were dissolved in 0.5% CMC-Na solution. Mice were administered LIN or SIL (intragastric administration, i.g.) once per day and CCl_4_ (intraperitoneal injection, i.p.; mixed 1:3 in olive oil, 2 mL/kg) twice per week for 4 weeks. Mice in the vehicle control group received olive oil (i.p. twice a week, 4 weeks) and 0.5% CMC-Na (i.g. every day, 4 weeks). After treatment, the mice were sacrificed, and plasma and liver tissues were collected and further analysed.

### 2.7 Biochemical parameters examination

Serum was collected from blood samples after centrifugation at 860 *×* g for 15 min. Serum ALT (Nanjing Jiancheng Bioengineering Institute, C010-2-1) and AST (Nanjing Jiancheng Bioengineering Institute, C009-2-1) activities were measured using kits, according to the manufacturer’s instructions. Liver hydroxyproline content was determined using the alkaline hydrolysis method, as described in the kits. Serum levels of laminin (LN) were measured using ELISA kits, according to the manufacturer’s instructions (Shanghai YANJIN Biological Technology Co., LTD, F12019).

### 2.8 Real-time PCR analysis

Total RNA was extracted from liver tissues and LX-2 cells using the TRIzol reagent. cDNA was synthesised using a PrimeScript RT Master Mix kit. Real-time PCR was performed using SYBR Green premix according to the manufacturer’s instructions. Relative expression of target genes was normalised to actin, analysed by the delta-delta-CT method and given as a ratio compared with the vehicle control. Primer sequences used in this study are listed in Supplementary Material.

### 2.9 Liver histological evaluation

A sample of the liver was fixed in 10% phosphate-buffered saline (PBS)-formalin solution and embedded in paraffin. Samples were sectioned (5 μm) and stained with haematoxylin-eosin (H&E) for histological observation of liver injury and stained with Masson’s trichrome and Sirius red to observe collagen deposition in the liver.

### 2.10 Immunofluorescence staining with α-SMA

The formalin-fixed and de-paraffinized liver sections (5 μm) were incubated with 5% bovine serum albumin to minimise non-specific binding, and then incubated with α-SMA antibody (Santa Cruz, SC-53142) in a humidified chamber overnight at 4°C. After washing thrice with PBS, the sections were incubated with FITC-conjugated secondary antibody (SH0058; Skyhobio) for 1 h. After washing thrice, the liver sections were incubated with DAPI for 10 min. Images were captured using an inverted microscope (IX81; Olympus, Japan).

### 2.11 Immunohistochemical staining

Paraffin-embedded liver sections were deparaffinized in xylene, rehydrated in a gradient of ethanol to distilled water, and then quenched by 3% hydrogen peroxide and then incubated with 5% bovine serum albumin, followed by incubation with vimentin antibody (ABclonal, A19607) at 4°C overnight, and further detected by using DAKO EnVision detection kits (Agilent Technologies Co., Ltd. K346811). The sections were counterstained with haematoxylin. Images were taken using an inverted microscope, and vimentin-positive cells were counted manually using Image-Pro Plus 6 in three random fields per sample (each group contained three samples).

### 2.12 Western-blot analysis

Liver proteins were isolated by using a lysis buffer containing 50 mM Tris-HCl (pH7.5), 150 mM NaCl, 1 mM EDTA, 20 mM NaF, 0.5% NP-40, 10% glycerol, 1 mM phenylmethylsulphonyl fluoride, 10 μg/mL aprotinin, 10 μg/mL leupeptin, and 10 μg/mL pepstatin. The supernatants were collected after centrifugation at 3,000 g for 10 min at 4°C, and the protein concentration of each sample was determined and normalised to the same protein concentration. Protein samples were separated by sodium dodecyl sulphate-polyacrylamide gel electrophoresis and transferred onto polyvinylidene fluoride membranes. The membranes were incubated with the indicated primary and secondary antibodies, and the proteins in the membranes were visualised using a chemiluminescence kit. TGF-^®^ antibody (CST, 3711), Smad 2/3 antibody (CST, 8685), p-Smad 2/3 antibody (CST, 8828), anti-α-SMA antibody (Santa Cruz, SC-53142), vimentin antibody (ABclonal, A19607), desmin antibody (ABclonal, A3736), GAPDH antibody (Beyotime, AF0006), and SHP2 antibody (CST, 3397). The grey densities of the protein bands were normalised using β-actin as an internal control, and the results were further normalised to the control.

### 2.13 Transcriptome sequencing and bioinformatics analysis

Total RNA was extracted using TRIzol reagent (Invitrogen, Carlsbad, CA, United States), according to the manufacturer’s protocol. RNA purity and quantification were analysed using a NanoDrop ND-1000 instrument (Wilmington, United States). RNA integrity was assessed using the Agilent 2100 Bioanalyzer (Agilent Technologies, Santa Clara, CA, United States). The libraries were constructed using the TruSeq Stranded mRNA LT Sample Prep Kit (Illumina, San Diego, CA, United States) according to the manufacturer’s instructions. Transcriptome sequencing and analysis were conducted by LC-Bio Technology Co. Ltd. (Hangzhou, China). The libraries were sequenced on an Illumina HiSeq X Ten platform, and 150 bp paired-end reads were generated. After generating the final transcriptome, the expression levels of all the transcripts were estimated. Differentially expressed mRNAs were selected using the following criteria: fold change >2 or fold change <0.5 and *p*-value <0.05. Hierarchical cluster analysis of differentially expressed genes (DEGs) was performed to determine the expression patterns of genes in different groups and samples. Gene Ontology (GO) enrichment and Kyoto Encyclopedia of Genes and Genomes (KEGG) pathway enrichment analyses of DEGs were performed using the R software based on the hypergeometric distribution.

### 2.14 Statistics and reproducibility

Quantitative data are presented as mean ± standard deviation (s.d.), as specified in the figure legends. Statistical tests were performed using GraphPad Prism 7.0. Two-sided Student’s *t*-tests were used to compare the means of the data between the two groups.

## 3 Results and discussion

### 3.1 Discovery of LIN as a novel SHP2 inhibitor from NP library

The recombinant human catalytic domain of the SHP2 protein (rhSHP2-PTP) was expressed in *E. coli* and purified using an affinity column, ion exchange column, and gel filtration. SDS-PAGE results showed the purified SHP2-PTP protein had a molecular weight of ∼37 kDa and a purity >95% (Figure 1A). DiFMUP was used as a fluorogenic phosphatase substrate. Enzyme titration experiments showed that rhSHP2-PTP had robust dephosphorylation activity toward the substrate DiFMUP (Figure 1B). Additionally, DiFMUP was tested at different concentrations to evaluate the catalytic kinetics of the dephosphorylation activity of SHP2-PTP (Figure 1C). Based on these linear enzyme and substrate concentration assay results, the SHP2-PTP and substrate DiFMUP concentrations were set to 0.5 nM and 10 μM, respectively, in the subsequent high-throughput screening (HTS) study (Figure 1D).

With the assay conditions established, an in-house focused NP library with 800 small molecules was screened in 384-well format. A total of eight initial hits exhibited ≥80% inhibition at a concentration of 20 μM, which were chosen for dose-response confirmation (Figure 1E). In a verification experiment of initial hits, furanogermacrane sesquiterpene linderalactone (LIN, at point 12I) showed the best inhibitory activity among the screened hits (Figure 1E).

### 3.2 Study on the analogues of furanogermacrane sesquiterpenes

*Lindera aggregata* (Sims) Kosterm is a common traditional herb that has multiple bioactivities (Ohno et al., 2005; Wang et al., 2022). A previous phytochemical study indicated that sesquiterpenoids may be the active components of *L. aggregate* (Wang et al., 2015). However, the bioactive molecules that contribute to its pharmacological activity and the underlying molecular mechanisms remain unknown. Our HTS results and the promising *in vitro* data from LIN encouraged us to further explore the SHP2 inhibition potency of sesquiterpenoid derivatives.

**FIGURE 2:**
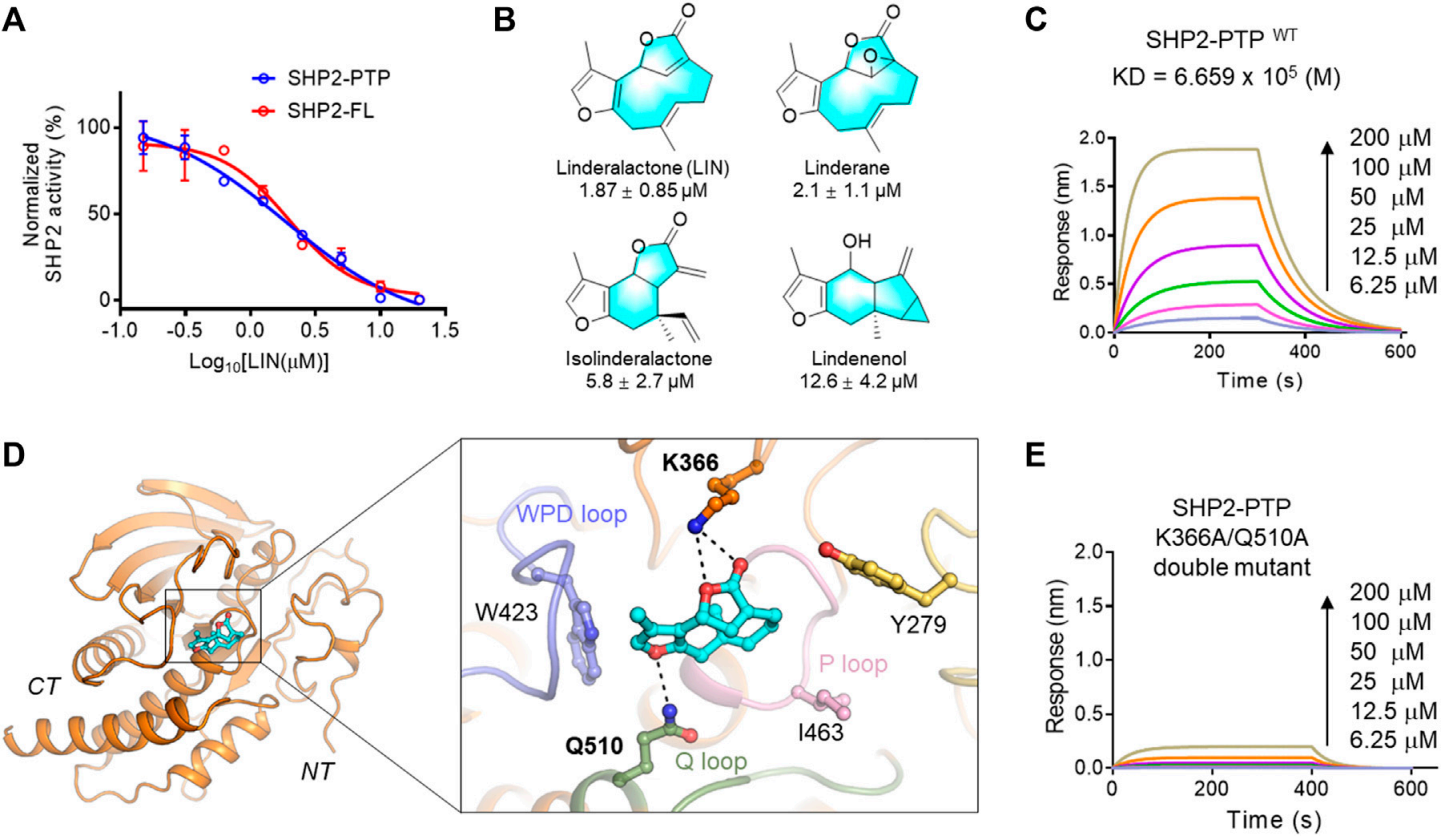
Validation of LIN derivatives as SHP2-PTP inhibitors. **(A)** IC_50_ of LIN on the enzyme activity of the full length (red line, SHP2-FL) and catalytic domain (blue line) of SHP2. Note that LIN showed a similar inhibition effect on SHP2-PTP and SHP2-FL enzymes with half-maximum inhibitory concentration (IC_50_) values at 1.87 and 1.98 μM, respectively. For full-length SHP2 assay, activating peptide p-IRS-1 was supplemented to active SHP2. **(B)** The chemical structures of LIN derivatives along with the SHP2-PTP IC_50_ values. **(C)** A bio-layer interferometry (BLI) assay evaluating the binding affinity of LIN to wildtype SHP2-PTP. **(D)** Binding mode analysis of LIN to SHP2 catalytic domain (PDB ID: 4RDD). **(E)** Binding affinity analysis of LIN to the SHP2^K366A/Q510A^ double mutant.

Linderane, isolinderalactone, and lindenenol, which have similar core scaffolds, were selected to test their SHP2-PTP inhibitory activity compared to that of LIN. Dose-response experiments of LIN showed an IC_50_ value of 1.87 ± 0.85 μM against the SHP2 PTP domain (Figures 2A, B). Linderane, isolinderalactone, and lindenenol, which were all incorporated with methylfuran group, displayed dose-dependent suppression of SHP2-PTP activity with IC_50_ values ranging from 2.1 to 12.6 μM, respectively (Figure 2B). Interestingly, compounds with an oxacycloundecin core (LIN and isolinderalactone) were more favourable for SHP2 inhibitory activity than linderane and lindenenol. Based on the *in vitro* enzyme assay results and its interesting novel structural skeleton, furanogermacrane sesquiterpene LIN was selected for further target and mechanistic studies.

### 3.3 Study on the SHP2 binding sites of LIN

As shown in Figure 2A, LIN showed a similar inhibitory effect on the enzyme activity of full-length SHP2, further demonstrating that LIN interacts with the PTP domain of SHP2. To generate more biochemical evidence to verify the direct binding with SHP2, the most active component, LIN, was selected to analyse the binding affinity with the PTP domain of SHP2 *in vitro* using a bio-layer interferometry (BLI) experiment. As shown in Figure 2C, LIN directly interacted with SHP2-PTP in a dose-dependent manner, with a K_D_ value of 665.9 μM.

To understand the underlying molecular basis of the LIN-SHP2 interaction, a molecular docking study was conducted using the PTP domain crystal structure of SHP2. The results revealed that LIN blocked the catalytic site of the PTP domain, thereby inhibiting the phosphorylated substrate from binding to SHP2. Predominantly, docking analysis indicated that residues Lys-366 and Gln-510 of the Q loop contributed to the LIN-SHP2 interaction by forming key hydrogen bonds with the two LIN furan fragments (Figure 2D). Additionally, the 3-methylfuran moiety of LIN forms hydrophobic interactions with the WPD loop of the SHP2 pocket, thus stabilising its binding to SHP2 and preventing the substrate from entering the active pocket.

To further validate the interacting residues and binding mode of LIN, K366A/Q510A double mutations were introduced into SHP2-PTP. As shown in Figure 2E, the K366A/Q510A mutation significantly eliminated the binding affinity between LIN and SHP2-PTP in the BLI experiment with a response value lower than 0.2 at the concentration of 200 μM. These experimental data facilitated the elucidation of the molecular mechanism by which LIN targets the key catalytic site of SHP2.

### 3.4 LIN attenuated CCl_4_-induced hepatic injury in mice

**FIGURE 3:**
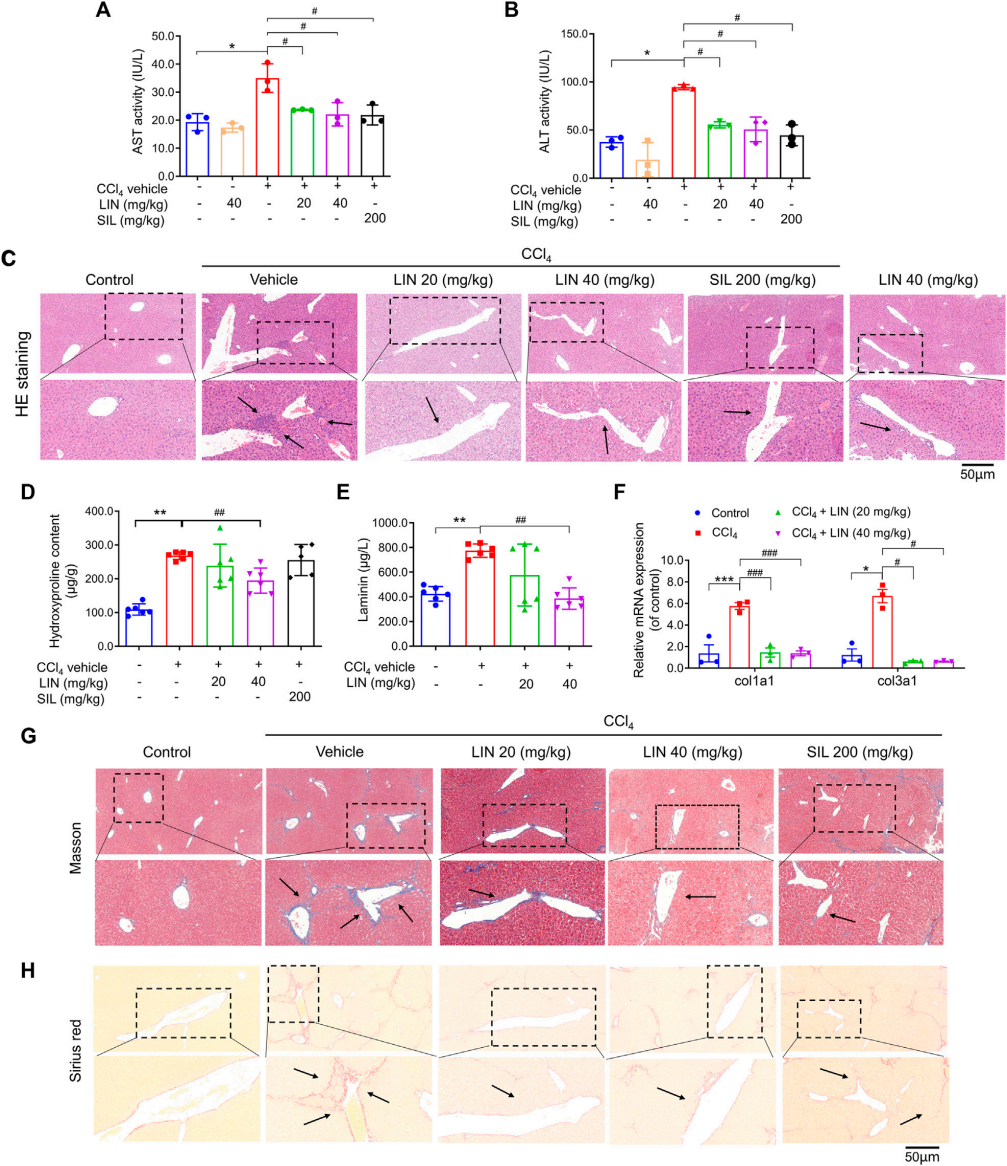
LIN attenuated CCl_4_-induced hepatic injury and fibrosis in mice. **(A)** AST activity. **(B)** ALT activity. **(C)** Liver H&E staining. Representative images were chosen from each experimental group. (Upper images: original magnification × 200; Lower images: partially enlarged pictures). Data were expressed as means ± SEM (*n* = 3).*p < 0.05 vs. control group; ^*#*^ *p* < 0.05 vs. CCl_4_ group. **(D)** Liver hydroxyproline content (*n* = 5–7). **(E)** Hepatic Col1a1 (Collagen, type I, α1) and Col3a1 (Collagen, type III, α1) (*n* = 3). **(F)** Serum contents of laminin (*n* = 7). **(G)** Liver Masson’s trichrome staining. Representative images were chosen from each experimental group. (Upper images: original magnification × 200; Lower images: partially enlarged pictures). **(H)** Liver Sirius red staining. Representative images were chosen from each experimental group. (Upper images: original magnification × 200; Lower images: partially enlarged pictures). Data were expressed as means ± SEM. *p < 0.05, **p < 0.01, ***p < 0.001 vs. control group; ^*#*^ *p* < 0.05, ^*##*^ *p* < 0.01, ^#*##*^ *p* < 0.001 vs. CCl_4_ group.

With promising *in vitro* results, the CCl_4_-induced liver fibrosis animal model was used to evaluate the therapeutic effect of LIN *in vivo*. Silymarin (SIL), a well-known hepatoprotective drug, was used as a positive control at a dose of 200 mg/kg. As shown in Figures 3A, B, serum AST/ALT levels were significantly increased in the CCl_4_ group, which is predictive of liver cell damage. Both ALT and AST levels were significantly reduced in the 20 and 40 mg/kg LIN-treated groups (*p <* 0.05), showing a protective effect similar to that of the 200 mg/kg SIL group. Histological analysis of the liver sections was performed to further evaluate the therapeutic effect of LIN on liver fibrosis. As shown in Figure 3C, CCl_4_-induced liver damage, including hepatic infiltration of immune cells, swelling, and necrosis of hepatocytes, was ameliorated by LIN treatment. In contrast, mice that received LIN alone at 40 mg/kg had no obvious alteration in ALT/AST and pathological changes in mouse liver tissue, indicating low LIN toxicity (Figures 3A–C). These findings indicated that LIN effectively attenuated CCl_4_-induced liver injury in mice.

### 3.5 LIN attenuated hepatic fibrosis in CCl_4_-induced mice model

Next, we investigated how LIN alleviated pathological changes in a mouse model of hepatic fibrosis. During liver fibrosis, the dynamic balance between synthesis and degradation of the extracellular matrix is disrupted, leading to ECM accumulation (including laminin, fibronectin, collagen I, III, and IV) and fibrosis formation (Biagini and Ballardini, 1989). Hydroxyproline is a characteristic fibrillar collagen component (Bradshaw et al., 2009). The antifibrotic effect of LIN was further evaluated by biochemical analysis of liver hydroxyproline and serum laminin levels. As shown in Figures 3D, E, hydroxyproline and laminin levels induced by CCl_4_ were decreased by LIN in a dose-dependent manner. Additionally, the increase in mRNA levels of *Col1a1* and *Col3a1* induced by CCl_4_ was significantly reversed after LIN treatment at a low dose (20 mg/kg) (Figure 3F).

Excessive accumulation of ECM is the major hepatic fibrosis pathogenesis, and collagen is considered to be the main ECM component. In the following study, two staining methods, Masson’s trichrome and Sirius red, were used to investigate the therapeutic effect of LIN on collagen deposition. Based on the results in Figures 3G, H, the CCl_4_-treated group showed disrupted hepatic architecture, increased collagen content, and bridging fibrosis. In contrast, LIN treatment ameliorated the extent of collagen deposition in a dose-dependent manner, especially in the 40 mg/kg LIN treatment group, which displayed mild collagen deposition in the liver without bridging fibrosis.

### 3.6 TGF-β/smad pathway is involved in the antifibrosis effects of LIN

**FIGURE 4:**
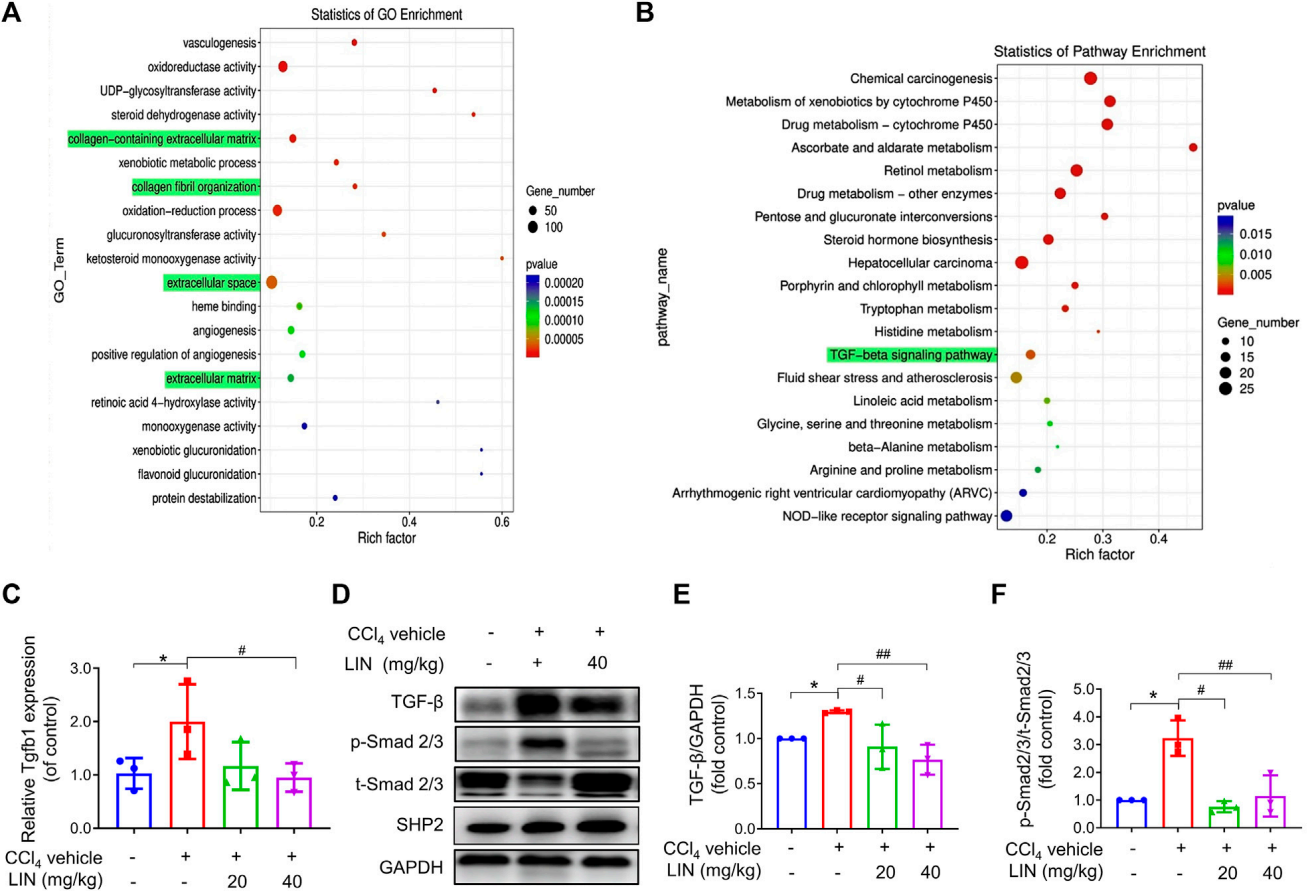
The anti-fibrosis effect of LIN is associated with inhibiting TGF-β/Smad signalling *in vivo*. RNA-seq showed 2163 differentially expressed genes with 716 genes upregulated, and 1447 genes downregulated between CCl_4_ and LIN + CCl_4_ groups, respectively (*n* = 3). RNA-seq showed 6480 differentially transcripts with 3450 transcripts upregulated, and 3030 transcripts downregulated between CCl_4_ and LIN + CCl_4_ groups, respectively (*n* = 3). **(A)** GO term enrichment analysis of differentially expressed genes revealed that LIN against CCl_4_ induces liver fibrosis possibly by regulating collagen−containing ECM and collagen fibril organization. **(B)** KEGG pathway analysis of differentially expressed genes showed that the potential mechanism of LIN regulating collagen−containing extracellular matrix was highly associated with the TGF-β signalling pathways involved. **(C)** Hepatic Tgfb1 (TGF-β) mRNA expression (*n* = 3). **(D)** The expression of liver TGF-β, p-Smad2/3, t-Smad 2/3, and SHP2 proteins was detected by Western-blot, and GAPDH was used as a loading control. **(E)** The quantitative result of TGF-β. **(F)** The quantitative result of p-Smad2/3. The results represent three independent experiments. Data were expressed as means ± SEM. *p < 0.05 vs. control group; ^#^ *p* < 0.05, ^*#*#^ *p* < 0.01 vs. CCl_4_ group.

To uncover the underlying mechanism of the antifibrotic effect of LIN, RNA sequencing analysis was performed using mouse liver tissue samples from the control, CCl_4_ group, and CCl_4_ + LIN groups. Among the 2163 DEGs, 716 genes were found to be upregulated, and 1447 genes were downregulated between the CCl_4_ and CCl_4_ + LIN groups. Additionally, 6480 differentially expressed transcripts with 3450 transcripts were upregulated and 3030 transcripts were downregulated. We used GO annotation analysis of DEGs to characterise their respective biological functions. Most biological-process-related genes between CCl_4_ and CCl_4_ + LIN groups were annotated with GO terms associated with “collagen−containing extracellular matrix”, “collagen fibril organization”, “extracellular space” and “extracellular matrix” (Figure 4A). Interestingly, based on KEGG annotation analysis, transforming growth factor beta (TGF-β) signalling emerged as the top 20 signalling pathways with statistical significance (Figure 4B), which plays a vital role in HSCs activation and ECM deposition that promote liver fibrosis.

We next investigated whether LIN prevented CCl_4_-induced liver fibrosis by intervening in the canonical fibrogenic TGF-β signalling pathway. Western blot and real-time-PCR results showed that the amplified mRNA and protein expression of TGF-β was reduced in mice treated with LIN (40 mg/kg) (Figures 4C–E). As shown in Figure 4F, Western blot analysis confirmed that the phosphorylation level of Smad 2/3 increased in the livers of CCl_4_-challenged mice, whereas LIN treatment significantly inhibited Smad2/3 phosphorylation in the liver. In addition, we checked the expression of SHP2 after LIN treatment (Figure 4D and Supplementary Figure S2). The result indicated that compound LIN do not affect the expression level of SHP2 protein, which demonstrated that SHP2 inhibitor LIN only inhibits the catalytic activity of SHP2. These results demonstrate that the SHP2 inhibitor, LIN, may alleviate liver fibrosis by interfering with the TGF-Smad3 pathway and HSCs activation.

### 3.7 LIN inhibited HSCs activation in CCl_4_-treated mice

**FIGURE 5:**
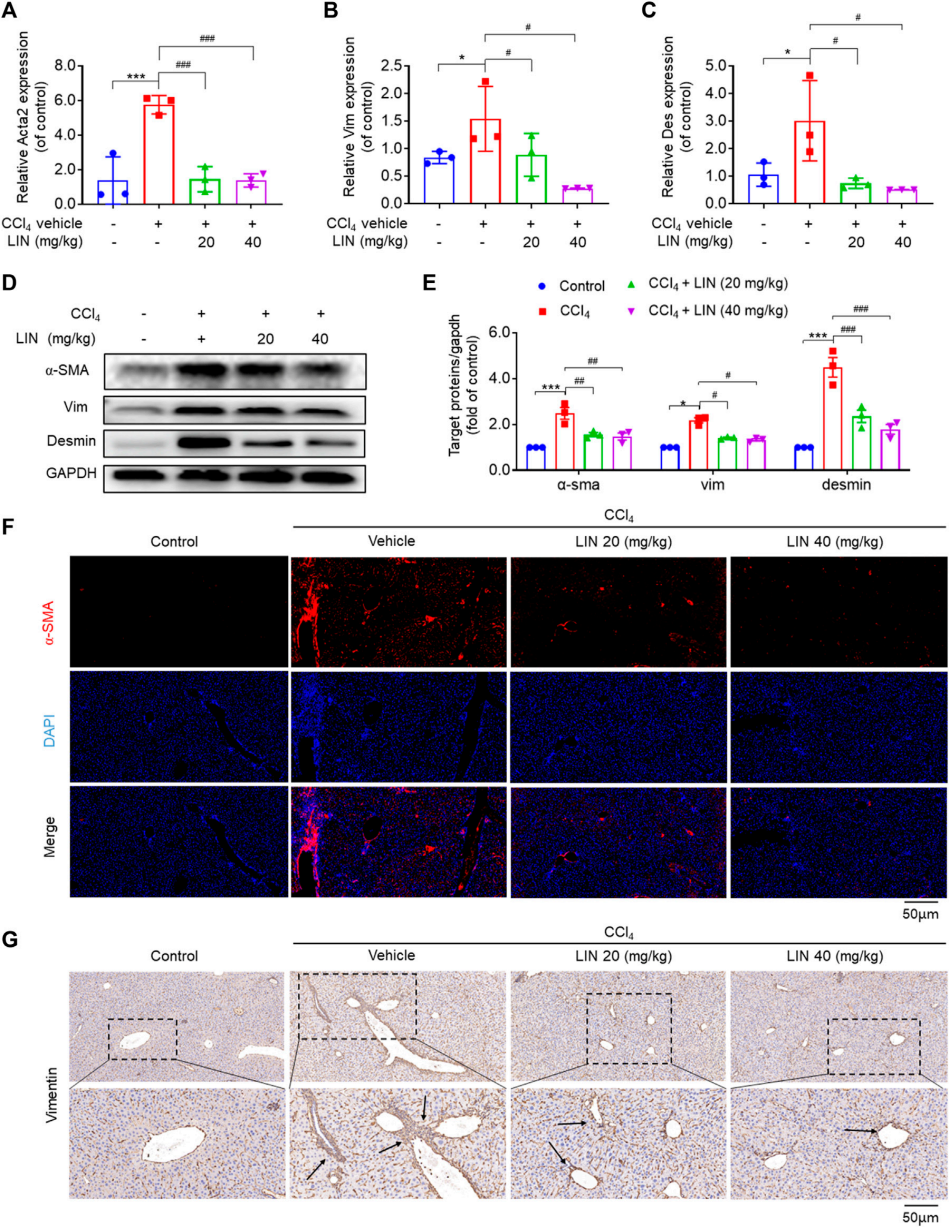
LIN inhibited HSCs activation in CCl_4_-treated mice. **(A–C)** Hepatic Acta2 (α-SMA), Vim (Vimentin), Des (Desmin) mRNA expression (*n* = 3). **(D)** The expression of liver α-SMA, vimentin, and desmin protein were detected by Western-blot, and GAPDH was used as a loading control. **(E)** The quantitative result of α-SMA, vim, and desmin. The results represent three independent experiments. **(F)** Liver α-SMA immunofluorescence staining (original magnification × 100). **(G)** Liver Vimentin immunohistochemical staining. Representative images are chosen from each experimental group. Data were expressed as means ± SEM. *p < 0.05, **p < 0.01, ***p < 0.001vs. control group; ^*#*^ *p* < 0.05, ^*##*^ *p* < 0.01, ^#*##*^ *p* < 0.001vs. CCl_4_ group.

Activated HSCs are recognised as the major matrix-producing cells during liver fibrosis progression. Several studies have indicated that SHP2 in HSCs contributes to process activation and fibrosis (Gao et al., 2020). Additionally, LIN may interfere with the TGF-Smad3 pathway based on RNA-sequencing analysis data; thus, we next investigated the effect of the identified SHP2 inhibitor LIN on HSC activation *in vivo*. Three well-known biomarkers, α-SMA, vimentin, and desmin, were used to investigate HSCs activation. data in Figures 5A–C showed that LIN (20 and 40 mg/kg) significantly reduced hepatic mRNA expression of *acta2*, *Vim*, and *Des* (*p* < 0.05, *p* < 0.01) induced by CCl_4_ *in vivo*. Treatment with LIN at a 20 mg/kg dose also caused a significant reduction in the protein expression of hepatic α-SMA, Vim, and Des (*p* < 0.05) (Figures 5D, E). Immunofluorescence staining (red) further confirmed that α-SMA accumulation was largely inhibited by LIN (Figure 5F). Additionally, a similar antifibrotic trend was observed in vimentin-positive cells after LIN treatment (Figure 5G).

### 3.8 LIN ameliorates hepatic stellate cell activation by inhibiting SHP2

**FIGURE 6:**
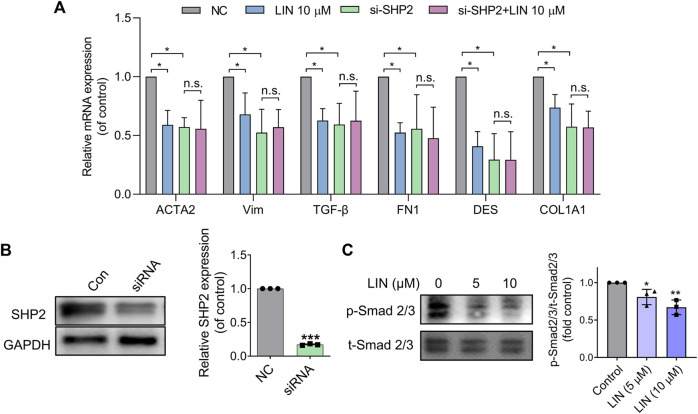
LIN suppresses HSCs activation by inhibiting TGF-β/Smad signalling. **(A,B)** LX-2 cells were transfected with control or SHP2 siRNA (20 pmol/1.0 × 10^5^ cells). Twenty-fourhours later, transfected LX-2 cells were treated with LIN at the indicated concentrations. Fibrogenic gene expression levels in LX-2 including *ACTA2*, *VIM*, *TGF-β*, *FN1*, *DES*, and *COL1A1* were evaluated by qPCR. SHP2 levels in siRNA-treated cells were confirmed by immunoblotting with anti-SHP2 antibody and qPCR. Experiments were performed in three biological repeats. **(C)** The expression of p-Smad2/3 and t-Smad 2/3 after LIN treatment (5 and 10 μM) was detected by Western-blot.

To confirm that the effect of LIN on HSC activation resulted from SHP2 inhibition, LX-2 cells were treated with 10 μM LIN for 24 h. As shown in Figure 6A, fibrogenic gene expression in LX-2 cells (e.g., *ACTA2*, *VIM*, *TGF-β*, *FN1*, *DES*, and *COL1A1*) was significantly decreased after LIN treatment, demonstrating the therapeutic potential of LIN to inhibit HSC activation.

To validate that the effect of LIN on LX-2 cell activation was mediated by inhibiting SHP2 activity, we further tested LIN in SHP2 knockdown cells. LX-2 cells were transfected with SHP2 siRNA and validated using western blotting and PCR (Figure 6B). As shown in Figure 6A, the mRNA expression levels of liver fibrosis markers were significantly suppressed compared to those in the control group, which is consistent with the positive role of SHP2 during fibrosis. Interestingly, SHP2-depleted LX-2 cells were much less sensitive to LIN treatment, suggesting that SHP2 may be the major target of LIN. Consistent with the *in vivo* result, LIN treatment led to significant reduced phosphorylation level of smad2/3 in LX-2 cells (Figure 6C).

## 4 Discussion

Liver fibrosis is the formation of a fibrous scar due to ECM accumulation, which replaces injured normal tissue (Friedman, 2003; Kisseleva and Brenner, 2021). During the past two decades, sustained progress has been achieved in the diagnosis and treatment of fibrotic liver disease. Non-etheless, currently, there are no approved drugs as effective therapeutic agents for liver fibrosis; thus, exploring new pharmacological therapeutic targets and drugs for liver fibrosis treatment is of great value.

SHP2 is a ubiquitously non-receptor PTP that was the first known carcinogenic PTP. SHP2 is involved in various vital signalling pathways, including JAK-STAT and RAS-MAPK caspases (Neel et al., 2003; Zhao et al., 2019; Marasco et al., 2020). SHP2 contributed to liver homeostasis maintenance by regulating inflammatory cytokine production (Liu et al., 2021). Recently, SHP2 was found to promote inflammation-driven insulin resistance, and pharmacological SHP2 inhibition in diabetic mice specifically reduced metaflammation and suppressed macrophage activation, thereby enhancing insulin sensitivity in mice (Paccoud et al., 2021). It has been reported that TGF-β1 can stimulate the phosphatase activity of SHP2, and SHP2 inactivation can repress TGF-β1 induced fibroblasts activation and relieve pulmonary and dermal fibrosis, indicating a positive role of SHP2 in fibroblast activation (Zehender et al., 2018). In the liver, the extracellular vesicles played an important role in liver fibrosis. SHP2 inhibition was found to reduce PDGFR enrichment in serum extracellular vesicles and alleviate liver fibrosis (Kostallari et al., 2018). This evidence offers new insights into the key role of SHP2 in inflammation and fibrosis. Thus, modulating SHP2 protein function has been considered an innovative potential therapeutic strategy for the intervention of tissue fibrosis.

Given its clinical significance, the discovery of novel SHP2 inhibitors for related diseases, such as liver fibrosis, is of great importance. To date, only a few SHP2 inhibitors have advanced to the early stages of clinical trials. Two types of SHP2 inhibitors have been developed. The first type of SHP2 inhibitor specifically binds to the PTP domain, thereby blocking enzymatic activity [e.g., NSC-87877 (Chen et al., 2006) and NAT6-297775 (Yuan et al., 2020)]. The second type of SHP2 allosterically stabilises its inactive conformation of SHP2. Several allosteric inhibitors, including JAB-3068 (Shen et al., 2020) and TNO155 (LaMarche et al., 2020) have advanced into clinical trials for cancer therapy. Recent studies indicate that currently developed allosteric inhibitors are ineffective against SHP2 gain-of-function mutants, such as E76K and D61V (Padua et al., 2018). Therefore, the search for SHP2 inhibitors with novel scaffolds to treat SHP2-mediated fibrosis is urgently required.

As the primary source of drug development, NPs are becoming increasingly important for their medicinal use in liver fibrosis therapy. Thus, identifying the molecular targets of NPs is essential for the discovery of novel antifibrotic agents. In this study, based on the established HTS SHP2 assay and natural products library, we identified a furanogermacrane sesquiterpene, LIN, as potential SHP2 active site inhibitors. DiFMUP and BLI assays validated that LIN directly binds to the SHP2 PTP domain. Further molecular docking and site-directed mutation experiments revealed that LIN binds mainly to Lys-366 and Gln-510 of SHP2 by forming key hydrogen bonds. Interestingly, among all the analogues, including linderane, isolinderalactone, and lindenenol, LIN showed the best potency against SHP2. This is the first report of the molecular targets of furanogermacrane sesquiterpene and LIN, which can be used as lead compounds or directly as candidate therapeutic agents to treat SHP2-related diseases. Since LIN functions as an orthosteric inhibitor by interacting with the substrate entrance of SHP2, LIN can potentially overcome drug-resistant gain-of-function mutants. Furthermore, SHP2 inhibition by LIN significantly inhibits liver fibrosis *in vivo*. The RNA-sequencing analysis further revealed that the SHP2 inhibitor LIN alleviates hepatic stellate cell activation by interfering with the TGF-Smad3 pathway. SHP2 deficiency in HSCs abolishes the anti-fibrosis effects of LIN. Thus, LIN or its derivatives could be considered potential therapeutic agents against SHP2-related diseases, such as liver fibrosis or NASH. Previous reports also indicated LIN could modulate the expression of apoptosis-related proteins and suppress the JAK/STAT signalling pathway (Rajina et al., 2020). And recent study indicated SHP2 increases STAT3 activation through JAK/STAT signalling (Fiebelkow et al., 2021). Thus, the effects of LIN on the roles of JAK/STAT pathway and cell apoptosis are worth to be further studies in the future.In summary, SHP2 activity is crucial in the process of liver fibrosis, and the search for novel drugs to inhibit the function of SHP2 has become a hot research topic in inflammatory and fibrosis-related diseases. Our study identified LIN as a structurally diverse scaffold candidate for liver fibrosis therapy. This novel active compound suppresses HSCs activation by inhibiting TGF-β/Smad signalling *in vivo*. Further structural modifications of LIN for the development of high-potency SHP2 inhibitors are currently underway in our laboratory.

## Data Availability

The original contributions presented in the study are publicly available. This data can be found here: PRJNA908299.

## Abbreviations

BLI, bio-layer interferometry; DEGs, differentially expressed genes; DiFMUP, 6,8-difluoro-4-methylumbelliferyl phosphate; ECM, extracellular matrix; HSCs, Hepatic stellate cells; HTS, high-throughput screening; IC50, half maximal inhibitory concentration; IPTG, isopropyl ß-D-1-thiogalactopyranoside; KEGG, Kyoto Encyclopedia of Genes and Genomes; LIN, Linderalactone; LN, laminin; NAFLD, non-alcoholic fatty liver disease; NPs, natural products; PTP, protein tyrosine phosphatase; RTK, receptor tyrosine kinase; SH2, Src homology 2; SHP2, Src homology 2 domain-containing phosphatase 2; SIL, silymarin; SSA, super streptavidin; TGF-β, transforming growth factor beta; α-SMA, α-smooth muscle actin.
