# Supplementary material for: Identification of linderalactone as a natural inhibitor of SHP2 to ameliorate CCl4-induced liver fibrosis
Source: Front Pharmacol. 2023 Feb 9;14:1098463. doi: 10.3389/fphar.2023.1098463 (PMC9946977; doi:10.3389/fphar.2023.1098463)
Supplement: Supplementary file 1 [file DataSheet1.docx]

Supplementary Material

**Table S1. List of Primers for Real-time PCR**

| Target | Full gene name | Gene ID | Primer | Sequence(5'-3') |
| --- | --- | --- | --- | --- |
| Col1a1 (mouse) | collagen, type I, alpha 1 | 12842 | FP | TGACTGGAAGAGCGGAGAGT |
|  |  |  | RP | GACGGCTGAGTAGGGAACAC |
| Tgfb1 (mouse) | Transforming growth factor, beta 1 | 21803 | FP | TGCCCTCTACAACCAACACA |
|  |  |  | RP | GTTGGACAACTGCTCCACCT |
| Acta2 (mouse) | actin, alpha 2, smooth muscle, aorta | 11475 | FP | GGGAGTAATGGTTGGAATGG |
|  |  |  | RP | GGTGATGATGCCGTGTTCTA |
| Col3a1  (mouse) | collagen, type III, alpha 1 | 12825 | FP | CTGGCCCTCCTGGTGCTTCT |
|  |  |  | RP | CCTTGGCCCATCCTTTCCTG |
| Vim  (mouse) | vimentin | 22352 | FP | CGGAAAGTGGAATCCTTGCAGG |
|  |  |  | RP | AGCAGTGAGGTCAGGCTTGGAA |
| Des  (mouse) | desmin | 13346 | FP | GCGGCTAAGAACATCTCTGAGG |
|  |  |  | RP | ATCTCGCAGGTGTAGGACTGGA |
| Actb (mouse) | actin, beta | 11461 | FP | TTCGTTGCCGGTCCACACCC |
|  |  |  | RP | GCTTTGCACATGCCGGAGCC |
| ACTA2  (human) | actin alpha 2, smooth muscle | 59 | FP | CTATGCCTCTGGACGCACAACT |
|  |  |  | RP | CAGATCCAGACGCATGATGGCA |
| VIM  (human) | vimentin | 7431 | FP | AGGCAAAGCAGGAGTCCACTGA |
|  |  |  | RP | ATCTGGCGTTCCAGGGACTCAT |
| DES  (human) | desmin | 1674 | FP | TCCAGTCCTACACCTGCGAGAT |
|  |  |  | RP | CGCAATGTTGTCCTGGTAGCCA |
| FN1  (human) | fibronectin 1 | 2335 | FP | ACAACACCGAGGTGACTGAGAC |
|  |  |  | RP | GGACACAACGATGCTTCCTGAG |
| COL1A1  (human) | collagen type I alpha 1 chain | 1277 | FP | GATTCCCTGGACCTAAAGGTGC |
|  |  |  | RP | AGCCTCTCCATCTTTGCCAGCA |
| ACTB  (human) | actin beta | 60 | FP | CACCATTGGCAATGAGCGGTTC |
|  |  |  | RP | AGGTCTTTGCGGATGTCCACGT |

FP, Forward Primer; RP, Reverse Primer


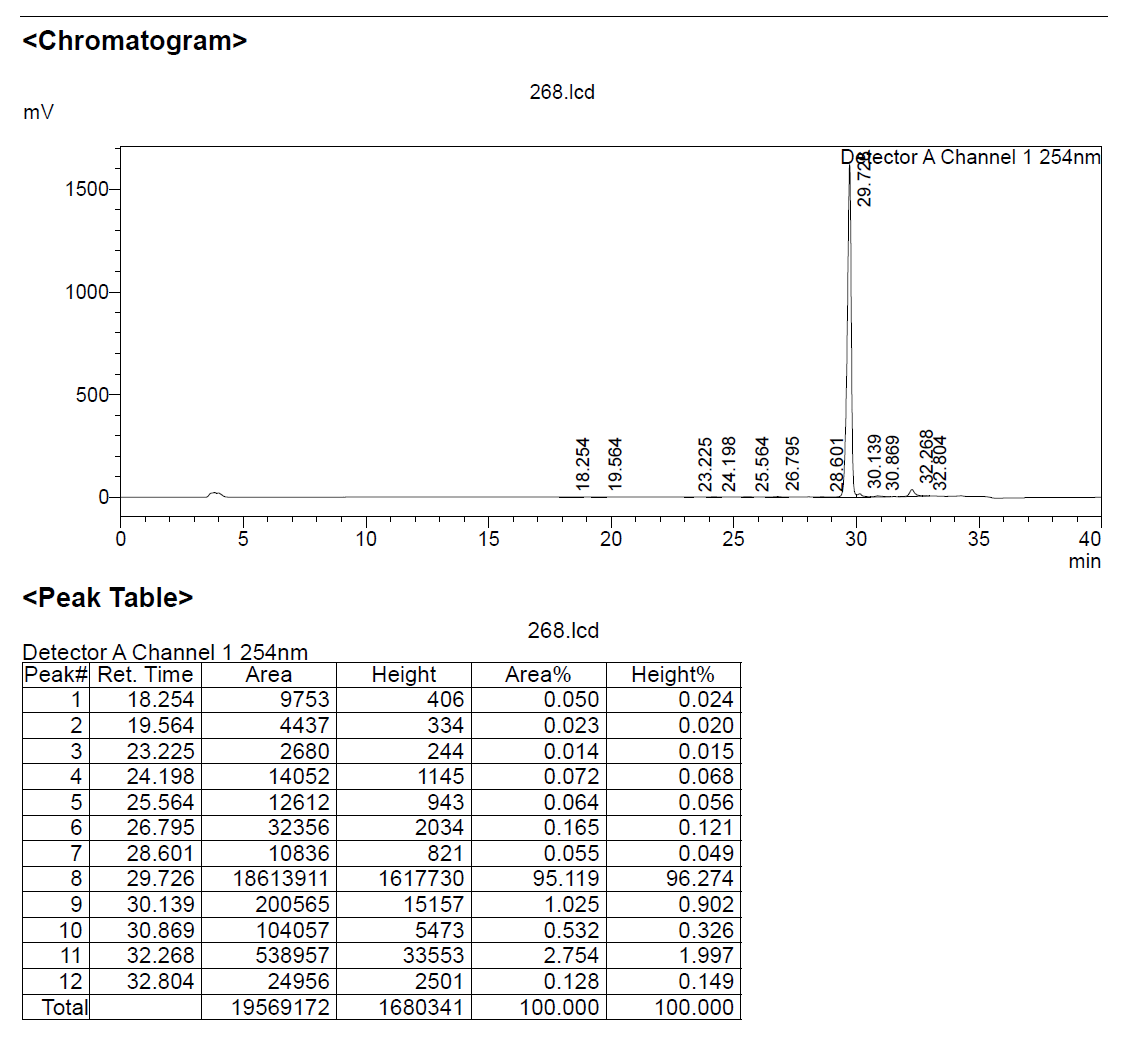


**Figure S1. HPLC traces for the compound LIN. The purity: 96.27%.**

**Figure S2. The expression of SHP2 after LIN treatment**


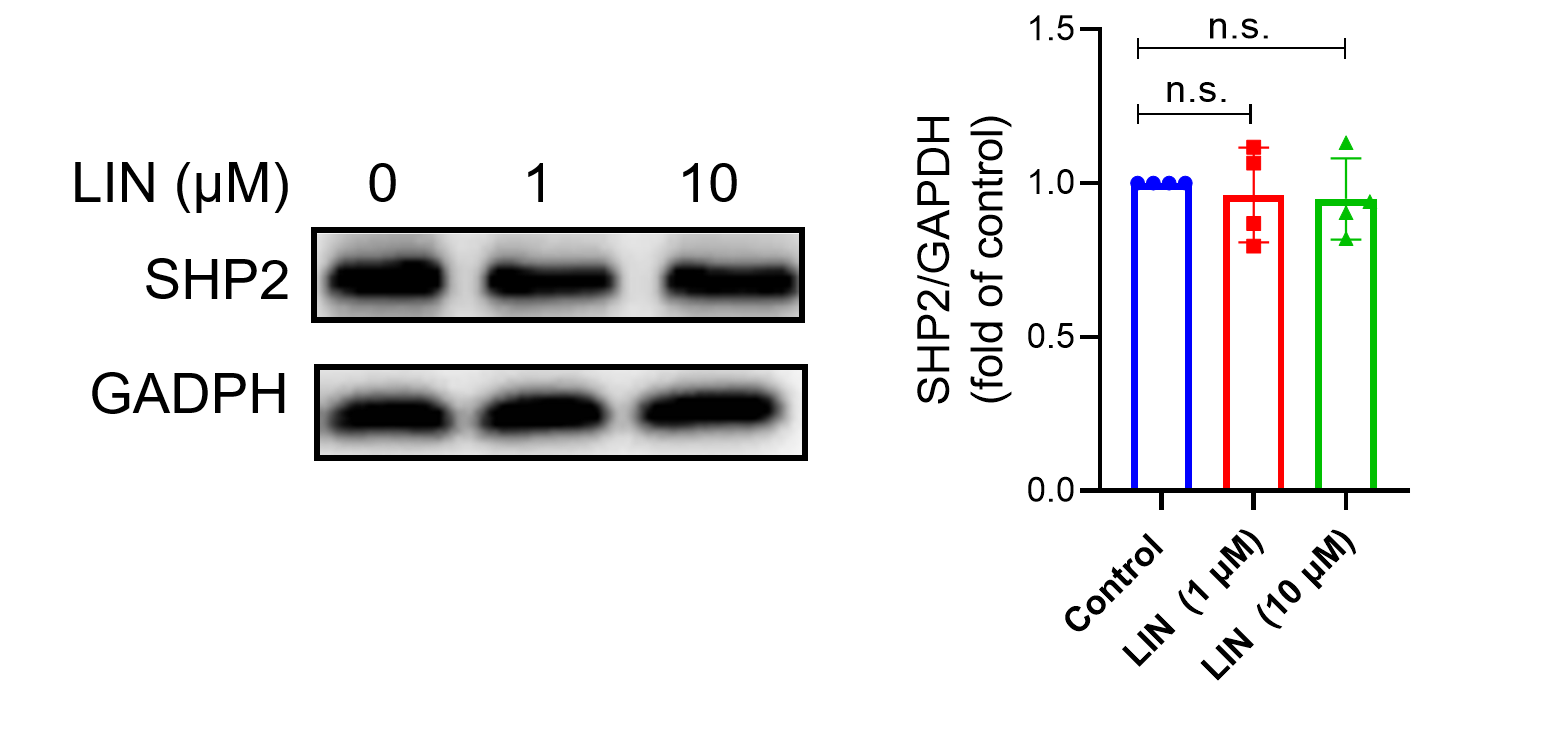


**Figure S2. The expression of SHP2 after LIN treatment.** LX-2 cells were treated with LIN at the indicated concentrations. SHP2 levels in cells were confirmed by immunoblotting with anti-SHP2 antibody. Experiments were performed in three biological repeats.
